# PBF-LB Process-Induced Regular Cavities for Lightweight AlSi10Mg Structures

**DOI:** 10.3390/ma14216665

**Published:** 2021-11-05

**Authors:** Victor Lubkowitz, Jonas Alber, Frederik Zanger

**Affiliations:** wbk Institute of Production Science, Karlsruhe Institute of Technology (KIT), Kaiserstraße 12, 76131 Karlsruhe, Germany; jonas.alber@kit.edu (J.A.); frederik.zanger@kit.edu (F.Z.)

**Keywords:** selective laser melting (SLM), process control, aluminum

## Abstract

In powder bed fusion with laser beam (PBF-LB), two process-induced defects by pore formation are known: local spherical pores by the keyhole effect and geometrically undefined pores caused by lack of fusion. Both pore types are heterogeneously distributed and can be used for lightweight or damping design applications. The achievable porosity is limited to around 13%. This article presents a novel process-controlled method enabling the targeted and reproducible manufacturing of solid parts with regularly distributed cavities, currently up to 60% porosity in AlSi10Mg, using the balling effect. This eliminates the need for time-consuming digital pre-processing work.

## 1. Introduction

Porous structures have been used successfully for many years in the form of metallic foams with porosities of around 50%. These are classically foamed during the casting process by admixing metal hydrides [1]. An attractive application is their use as the cores of structural sandwich panels to achieve good shear and fracture strength at a low weight [2]. In addition, foams are used for energy absorption as they allow large deformations at almost constant stress and have great damping properties due to their high number of gas- or powder-filled cavities [3]. They are well suited as fine filters due to their good particle retention in combination with the mechanical properties [4]. Depending on the material, they are very suitable in the food industry and for medical applications [5]. Considerable potential is seen in the production of patient-specific, porous, structured implants by additive manufacturing [6]. Numerous approaches exist to produce mathematically calculated irregular foam-like or regular lattice structures using predefined unit cells. A strong disadvantage of them is the high computational cost required for meshing and mapping large numbers of units, making CAD files hard to operate with [7]. For this purpose, it is favorable to be able to generate process-related pores in a stable and regular manner simply by choosing process parameters without additional computations. 

The aim of the investigations presented in this paper was to control the laser powder bed fusion (PBF-LB) process parameters to adjust the porosity in a targeted manner without the need for a complex design. The maximum achievable porosity caused by keyholing and lack of fusion was investigated. Furthermore, the generation of three-dimensional cavities by significantly increasing the layer thickness up to 600 µm and by the targeted use of known weld track formations was examined. Therefore, humping and balling weld tracks are of particular interest.

## 2. Mechanisms of Pore and Weld Track Formation

PBF-LB is one way of producing metallic additive components. It allows the producing of individual parts with locally varying properties. It is based on the layer-wise fusion of metal powder by a laser source. Weld tracks are laid side by side to create the component. 

Phenomenological studies have led to material-dependent process parameters that ensure a relative density above 99.7%. The process parameters of laser power (P_L_), scan velocity (v_s_), hatch distance (h_s_), and powder-layer thickness (l_s_) are adjusted to avoid the two known pore generating defects, the keyhole effect and lack of fusion. Often, the influencing process parameters are summarized to the energy density (E_V_) to show the influences on the process in a simplified way. A rarely used alternative is the calculation of the line energy density (LED) based on the values of laser power and scan velocity:

E_V_ = P_L_/(v_s_ · h_s_ · l_s_) (1)

LED = P_L_/v_s_ (2)

**Process-pores by keyhole effect:** Increasing laser power, as well as decreasing scan velocity, leads to an increased energy density. When the energy density is significantly increased, compared to the energy density required to produce pore-free layers, the metal will vaporize. Simulations of TiAl6V4 show that the vaporization results in recoil pressure, which creates a cavity inside the melt pool. The high laser power further causes the keyhole bottom to superheat, resulting in a drop of the surface tension compared to the keyhole neck. The stress difference causes the keyhole to constrict, resulting in the detachment of spherical bubbles. By repeating this effect, the melt pool behind the current processing zone is set into rotation, causing the bubbles to disperse in the melt. As most of these gas bubbles do not rise from, but remain in, the liquid melt zone, they cause voids in the solidified material [8]. The formation of process pores is also investigated through a process of high-speed X-ray imaging to take a detailed look at the keyhole formation in a titanium alloy [9]. 

**Lack of fusion:** Laser power and scan velocity have a decisive influence on the geometric shape of the melt pool. When the power is significantly reduced or the scan velocity significantly increased, the energy input causes a heat-conduction welding process rather than a deep melt pool. Consequently, the melt pool penetrates less deeply into the previous layer or only touches it, which reduces the adhesion of the layers to each other. This leads to the formation of open, undefined pores. The same applies if the hatch distance is too large. The individual weld tracks lie next to each other and do not weld together, resulting in the same type of pore [10].

Both of the explained defect types can be exploited by specific and local adaptation to produce foam-like structures for functional integration. An investigation analyzing the effect of sub-optimal density on the mechanical properties of AlSi10Mg worked with a maximum porosity of 4% [11].

However, the maximum achievable porosity that can be set with both methods has not yet been investigated. Other research has attempted to produce porous structures by using alternative printing parameters, increasing the hatch distance to produce separated, thin walls close to each other. In that case, solid walls were produced, only suitable for macroscopic structures [12]. The influence of layer thickness, varied in a normal range between 25 and 45 µm for the PBF-LB process, energy density, and laser power on porosity, hardness, and surface quality has been described in another investigation. A porosity of ~30% in the material 316 L was observed by a lack of fusion with low energy density and high layer thickness [13].

A new method to adjust the porosity can be the targeted utilization of the various weld track shapes that are formed depending on the process parameters. 

Weld track formations: A distinction is made between the solid, humping, and balling formations. A high energy density results in a straight, solid weld track with clear edges, fully diluted into the substrate. Through a reduction in the energy density, analogous to the laser welding process, the weld track starts humping because of the surface tension effect and a turbulent melt pool flow induced by vapor pressure, similar to the previously mentioned keyholing conditions. Small parts of the weld track start to tear and disconnect. The weld track starts warping [14,15,16].

Further energy reduction leads to balling. Low laser power and high scanning velocities result in a lack of dilution with the substrate. The lacking connection of the weld track with the substrate and a high scan velocity separate the track into smaller sections. To reduce the free surface energy, small, spherical balls form [14].

## 3. Provocation of Process Pores

In two CCD test plans, the factors of the laser power, scan velocity, and hatch distance were varied to analyze the degree of porosity that can be stably generated by inducing fusion or process pores. For this purpose, three cylindrical samples per set of parameters, with a diameter of 10 mm and a height of 12 mm, were set up with AlSi10Mg powder, which had a particle-size distribution (PSD) of 20–63 μm. The test pieces were built on a SLM280 HL 1.0 from SLM Solutions with a maximum laser power of 400 W, utilizing a laser wavelength of 1070 nm, and a scan velocity between 200 and 3000 mm/s. The building plate was pre-heated to a temperature of 473.15 K. Subsequently, the density was determined (using the Archimedes’ principle with distilled water), and the pore distribution as well as the shape were observed by optical inspection of microsections. An ideal density of 2.68 of dense AlSi10Mg was used to calculate the porosity.

### 3.1. Process Pores 

**Figure 1:**
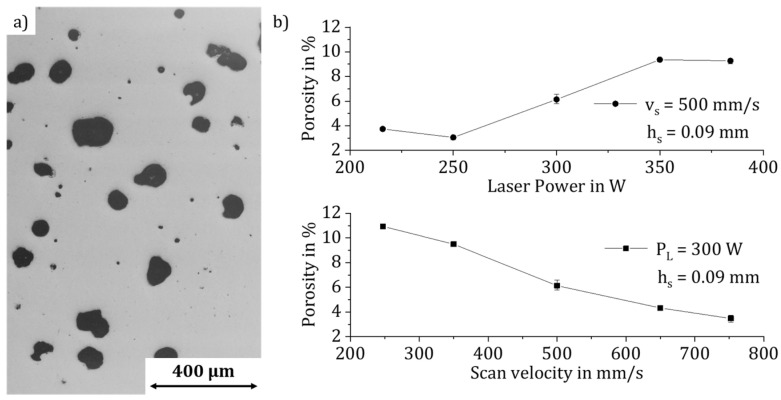
Influence of the main parameters P_L_ and v_s_ on the formation of process pores in the material AlSi10Mg: (**a**) microsection, (**b**) influence of the process parameters.

At first, the energy density was significantly increased from the material’s standard 35 J/mm^3^ to 300 J/mm^3^. Nevertheless, only irregularly distributed spherical process pores (Figure 1a, evaluation of microsections) and an overall porosity of 11.1% could be reached; see Figure 1. The significant influence of laser power and scan velocity on the keyhole effect is confirmed by a Pareto effect analysis. The effect of the hatch distance on the porosity is negligible. With increasing laser power and decreasing scan velocity, more pores can be generated in the compound. The density determination throughout the test specimens proves that process pores can be adjusted in a stable and reproducible way as the standard deviation is below 0.43%; see Figure 1b.

### 3.2. Lack of Fusion Pores

**Figure 2:**
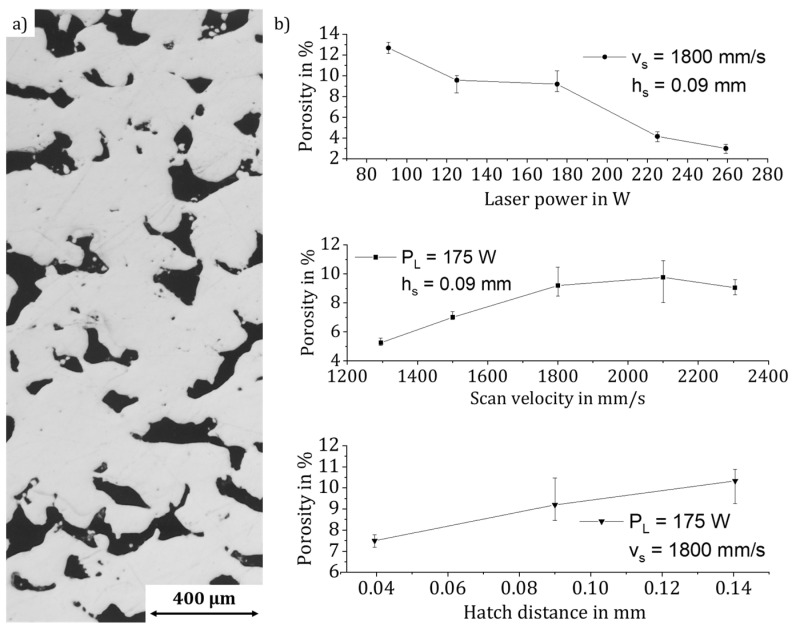
Influence of the main parameters P_L_, v_s_, h_s_ on the formation of lack of fusion pores in the material AlSi10Mg: (**a**) microsection, (**b**) influence of the process parameters.

Secondly, the energy density was significantly reduced to 4 J/mm^3^, resulting in a maximum stable density reduction of 12.9%, which is slightly higher than the one introduced by the keyholing effect. Again, the significant influence of the parameters of the laser power, scan velocity, and hatch distance on the formation of fusion pores is confirmed by a Pareto effect analysis. The influence of the factors corresponds to the state-of the-art level of the fusion pores; see Figure 2. The slightly higher deviation of up to 1.93% results from the difficulties in measuring the density determination of these open-pored parts.

### 3.3. Description and Linking of the Results by the Line Energy

**Figure 3:**
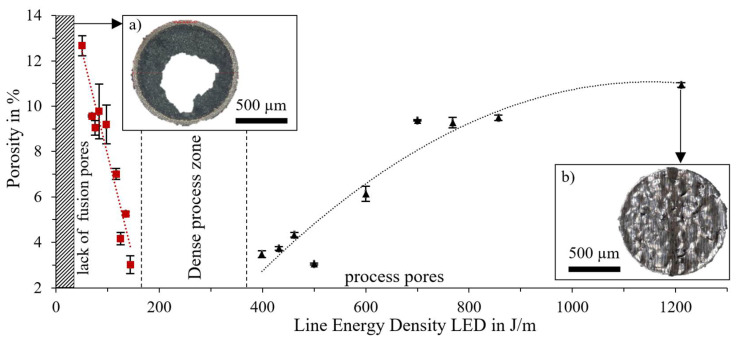
Definition of process windows as a function of LED to describe the porosity as a result of the pore formation mechanisms: (**a**) representation of the process limit at the lowest LED due to unstable structures, (**b**) representation of geometric surface deviations due to melt pool turbulence.

Three process windows can be determined by representing the porosity from the effects process pores or lack of fusion as a function of the line energy density. At low LED, porosity is formed due to a lack of fusion. The change in density can be described by a linear equation. A further reduction in LED to further increase the porosity is not feasible. Due to insufficient layer adhesion in the heat-conduction welding process, the structures collapsed, so that no coherent samples could be built up; see Figure 3a.

At high LED, porosity results from the process pores. The porosity reaches a maximum at maximum LED values. A further increase in density through this effect is hence not possible. Due to the high laser power and low scan velocity, the weld track is locally in a molten state for longer periods of time, which gives the enclosed inert gas bubbles more time to rise to the melt pool surface and thus not remain behind in the sample. The process pores move upwards due to the rolling movement of the melt pool as described by Bayat et al. in [8]. A clear deformation of the sample surfaces at high LED is an indication of the unsteady melt pool; see Figure 3b.

To produce dense materials, the LED has to be set to moderate values, as indicated in Figure 3 (“dense process zone”).

However, both effects do not allow a homogeneous local distribution of the pores, and the maximum achievable porosity for the material AlSi10Mg is limited to 12.9%. A new process strategy is required to increase the porosity.

## 4. Novel Production of Cavities Using Melt Tracks

As mentioned previously, unit cells are currently state-of-the-art in the production of parts with a low density. A novel method is needed to lower the model complexity and, linked to this, the required time in the pre-process when computing the CAD data and transforming it into machine code. This is enabled by the creation of process-controlled three-dimensional structures, which consist of solid parts and cavities.

### 4.1. Experimental Work

**Figure 4:**
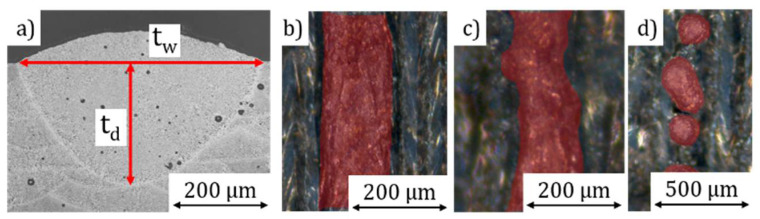
Analysis of the melt tracks produced with different LED: (**a**) microsection measurement, (**b**) stable, (**c**) humping, and (**d**) balling weld track.

The impact of the process parameters on the weld track formation must be known to be able to adjust the porosity precisely. Twenty-millimeter-long weld tracks were produced with different laser power (100 to 385 W), scan velocity (300 to 3000 mm/s) and powder-layer thickness (50 to 600 µm) to investigate the possible maximum depth with viable connection between the test specimens and the weld track. Subsequently, the shape of the tracks was analyzed with macroscopy and categorized in four different categories: stable track, humping, balling, and nonexistent. Selected track depth (t_d_) and width (t_w_) were measured by microsections and CT-scans; see Figure 4.

Based on the results, cubes with an edge length of 10 mm with different cavities were produced. The rotation of the weld track orientation between each layer was set to 90° and the hatch distance was varied. The cubes were then examined in the same way as the weld tracks.

### 4.2. Weld Track Formation

**Figure 5:**
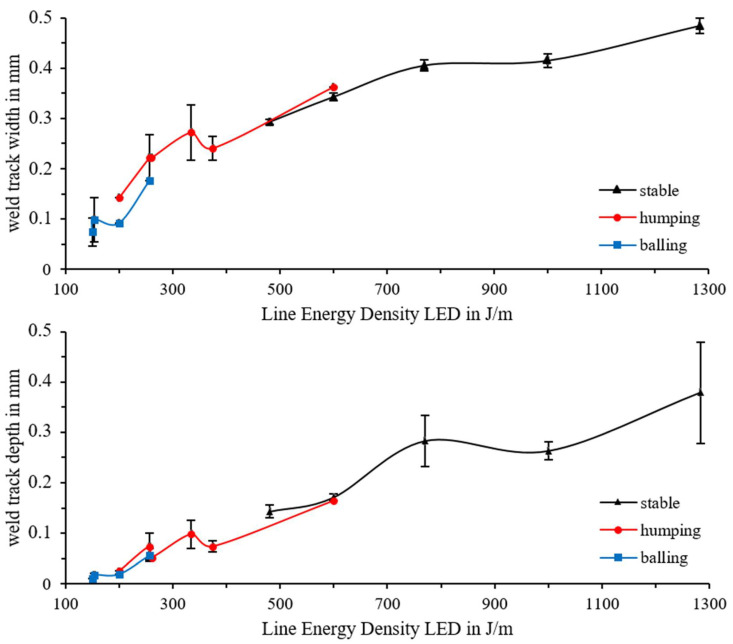
Weld track width and depth vs. scan velocity for two different values of laser power.

The track width (t_w_) increases with an increased LED while the layer thickness does not influence the weld track width significantly. The decisive factors for the width of the weld track are the shape and the applied line energy density. At low LED, a small melt pool forms that adheres only to the surface of the test specimens and contracts into small balls to reduce the free surface energy. Therefore, a small contact width of the balls on the surface of the test specimen is measured. With increasing LED, the weld track width increases, and a small weld root is formed in the test specimen. Humping weld tracks are created. A further increase in LED leads to a fully formed weld root and stable weld tracks. In the LED ranges between ~200 and ~250 J/m and ~375 and 600 J/m, respectively, a change in weld track shape occurs. Between the ranges of clear allocation to one of the four categories are small transition ranges with superimposed characteristics. The increase in the width of the weld track with increasing LED is attributed to the increasing dimensions of the melted material due to the increasing weld pool size; see Figure 5. The results are generally consistent with those of Aboulkhair et al. who observed the same effect of weld track width as a function of shape [17].

Measurements of the weld track depth show similar results. Higher LEDs result in a higher weld track depth due to a deeper keyhole/melt pool depth. The rise in the weld track depth can be explained by the keyhole effect and rising vapor depression depth, as Martin et al. have shown [18]. The amount of process-pore occurrence also increases due to a stronger keyhole effect. The increasing variation in weld track depth can also be attributed to the formation of the process pores. Due to the repeated contracting of the keyhole, the melt pool depth reduces before the keyhole is fully formed again, as described by Bayat et al. [8]. By measuring the weld track width in the microsections, it is only measured at one point, from which the variation is derived; see Figure 5. 

**Figure 6:**
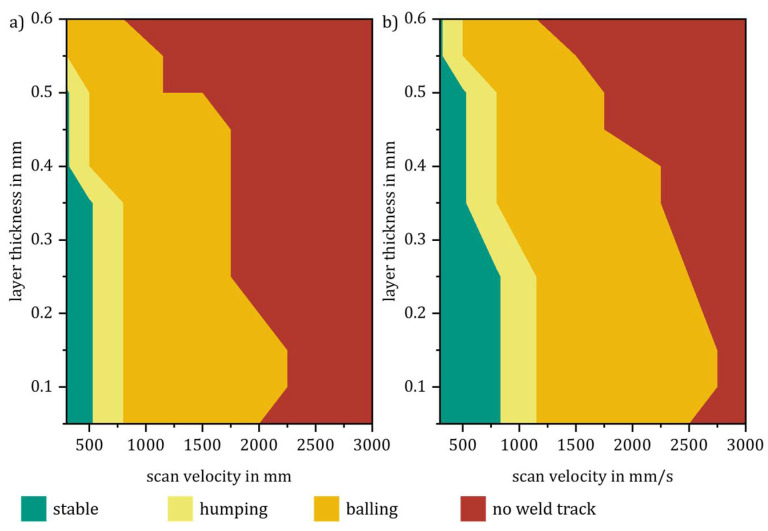
Weld track categories vs. scan velocity, layer thickness, and laser power: (**a**) P_L_ = const. 300 W, (**b**) P_L_ = const. 385 W.

The results shown in Figure 5 are also reflected in the categorization of the weld tracks. With the increasing line energy density, due to higher laser power and lower scan velocity, and therefore a deeper melt pool zone, a stable range of weld tracks with higher layer thicknesses can be produced. In the same way, the range of stable weld tracks (green), humping (yellow), balling (orange), and no weld tracks (red) also shift; see Figure 6.

### 4.3. Production of Regular Cavity Volume Test Specimens

Based on the results of the weld track investigations, different structures can be built. To produce three-dimensional porous structures, only the stable weld track parameters were used. The structures can be separated into three types: sinter, lattice, and spherical. 

On top of the discussed parameters (laser power, scan velocity, and layer thickness), for the two-dimensional weld tracks a fourth parameter is crucial to create three-dimensional structures: the lattice gap (g_w_). It represents the cavity size better and will therefore be used to characterize the new structures. The lattice gap is defined as:

g_w_ = h_s_ − t_w_ (3)

The porosity of the volume specimens presented in the following was derived from the analysis of the recorded CT images. The Archimedes’ principle used with the former specimen is not suitable, due to open structures and a liquid entry of the distilled water.

If the lattice gap is set to less than 0.1 mm and the layer thickness below 0.2 mm, sinter-like structures are formed. The agglomerates adhering to the melt pool of the weld tracks prevent complete powder removal. A porosity of around 40% can be achieved. 

**Figure 7:**
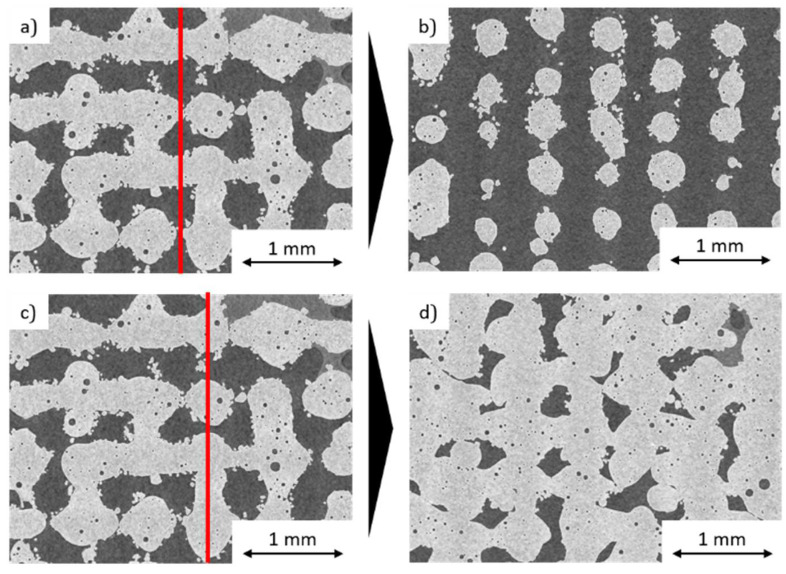
CT-Image: (**a**) top view, (**b**) sectional side view, (**c**) top view, (**d**) sectional side view of a lattice-structure (P_L_ = 385 W, v_s_ = 500 mm/s, g_w_ = 0.3 mm, l_s_ = 250 µm).

Increasing the lattice gap to 0.3 mm and 0.5 mm and the layer thickness incrementally from 0.05 mm to 0.3 mm results in a distinctive lattice structure. The weld tracks lose their bond to the previous layer as soon as the layer thickness is higher than the melt pool depth. This results in a three-dimensional lattice. Humping occurs between the intersections, due to the missing connection of the weld track to the previous layers. This effect can be seen clearly in the CT examinations. A CT plane analysis of the lattice structure was performed at two locations. The first plane is placed between the intersection points of the weld lines and shows the formation of round bars of the lattice that are not connected to each other; see Figure 7a,b. The second plane cuts through the intersection points of the weld tracks and shows a good adhesion of the individual weld tracks to each other; see Figure 7c,d. One advantage of these open structures is their potential to be completely de-powdered. A porosity of 55% can be achieved. Increasing the gap size would increase the porosity even further but is limited to the point where the welding tracks completely constrict. However, the limit of the hatch distance has yet to be determined.

**Figure 8:**
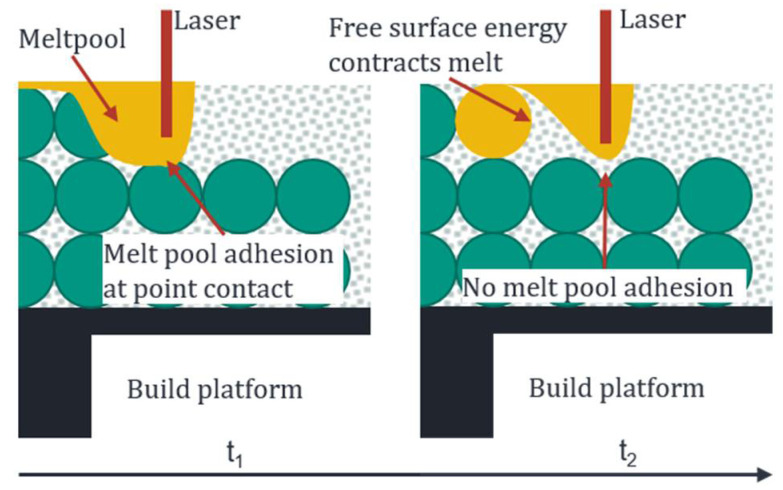
Explanation of the spherical-structure creation process.

Increasing the layer thickness minimally higher than the weld track depth, from 0.35 mm to 0.55 mm, allows the production of spherical structures with up to 60% porosity. When the weld tracks are produced, there is only a point of contact between the molten bath and the underlying track at the crossing points. The low adhesion results in the strong humping and balling of the weld track. To dissipate the free surface energy, the melt pool contracts into balls when the melt pool moves on and loses connection to the structure; see Figure 8. When slicing the corresponding structures, it must be considered that the top layer corresponds to the n-1 layer of the powder bed as the weld tracks do not build up but sink completely into the powder bed. 

**Figure 9:**
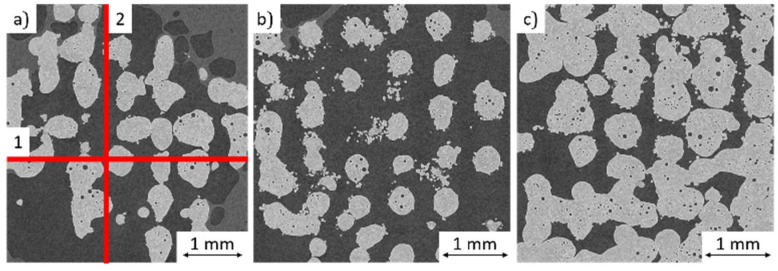
CT Image: (**a**) top view, (**b**) plane 1 sectional side view, (**c**) plane 2 sectional side view of a spherical structure (P_L_ = 300 W, v_s_ = 300 mm/s, g_w_ = 0.3 mm, l_s_ = 350 µm).

The CT images of the spherical structures show that the connection between the individually forming spheres is minimal or currently partially missing, making the structures unstable; see Figure 9. Thus, stabilization of the process is still necessary to ensure point contact everywhere between the spheres for a significant strength.

## 5. Conclusions

A new method to produce cavities in the PBF-LB process was investigated and presented. It was proven that for AlSi10Mg the porosity cannot be increased above 12.9% by inducing pores with known defect mechanisms. With the variation of the process parameters, it is possible to produce process-controlled cavities with up to 60% porosity. This offers the possibility to use the advantages of metallic foams locally and targeted to additive manufacturing for lightweight constructions without the need of complex model data, reducing computing time considerably.

The main results of the presented research are:

- Process pores from the keyhole process form as spherical bubbles irregularly distributed in the component. The maximum stable porosity generated is limited to 11.1%.
- Fusion pores form as irregularly distributed open pores. The maximum, stable, adjustable porosity is limited to 12.9%.
- By setting the process parameters accordingly, three-dimensional lattice structures can be produced, reaching up to 55% porosity.
- The creation of spherical structures increases the potential porosity up to 60%.
- The formation mechanisms of the types of pores and the generation of cavities can be fully described, making them transferable to other materials with manageable effort.
- The new approach can significantly reduce complex and time-consuming pre-processing.

In further investigations, the generation of spherical structures must be stabilized and the hatch distance limit for lattice structures must be evaluated.

## Data Availability

The data presented in this study are available on request from the corresponding author after obtaining the permission of an authorized person.
